# Identification of gene interactions associated with disease from gene expression data using synergy networks

**DOI:** 10.1186/1752-0509-2-10

**Published:** 2008-01-30

**Authors:** John Watkinson, Xiaodong Wang, Tian Zheng, Dimitris Anastassiou

**Affiliations:** 1 Center for Computational Biology and Bioinformatics and Department of Electrical Engineering, Columbia University, 500 West 120th Street, New York, NY 10027, USA; 2 Department of Statistics, Columbia University, 1255 Amsterdam Avenue, New York, NY 10027, USA

## Abstract

**Background:**

Analysis of microarray data has been used for the inference of gene-gene interactions. If, however, the aim is the discovery of disease-related biological mechanisms, then the criterion for defining such interactions must be specifically linked to disease.

**Results:**

Here we present a computational methodology that jointly analyzes two sets of microarray data, one in the presence and one in the absence of a disease, identifying gene pairs whose correlation with disease is due to cooperative, rather than independent, contributions of genes, using the recently developed information theoretic measure of synergy. High levels of synergy in gene pairs indicates possible membership of the two genes in a shared pathway and leads to a graphical representation of inferred gene-gene interactions associated with disease, in the form of a "synergy network." We apply this technique on a set of publicly available prostate cancer expression data and successfully validate our results, confirming that they cannot be due to pure chance and providing a biological explanation for gene pairs with exceptionally high synergy.

**Conclusion:**

Thus, synergy networks provide a computational methodology helpful for deriving "disease interactomes" from biological data. When coupled with additional biological knowledge, they can also be helpful for deciphering biological mechanisms responsible for disease.

## Background

The problem addressed in this work is the inference of gene-gene interactions that are specifically associated with a phenotype (such as a particular cancer) from two sets of gene expression data, one in the presence and one in the absence of the phenotype, and without use of prior biological knowledge. This problem is fundamentally different from that of inferring gene-gene interactions from one set of microarray data, for which several techniques have been proposed [1], such as those based on Bayesian networks [2,3], pairwise mutual information [4,5] and graphical Gaussian models [6,7]. In our case, any interactions representing general biological functions that are unrelated to the phenotype are ignored. Coupled with additional biological knowledge, the identification of such phenotype-specific interactions has the potential of shedding light on the responsible pathways. The term "cancer interactome" has been used in the above context, and part of the aim of this paper is to provide a novel methodology that is helpful for the derivation of such interactomes.

To solve this problem, we may wish to apply a traditional gene interaction network inference methodology, such as Bayesian network inference, on each of the two microarray data sets, for example one representing healthy samples (tissues) and another representing cancerous samples, and then compare the two resulting networks (the "normal" network and the one that has been "rewired" due to the disease) in an effort to identify differences in gene membership and network topology that may be related to the phenotype. However, each of the two resulting networks will be affected in different ways depending on the nature and number of the samples in each category. Furthermore, constructing the topology of network graphs often requires the use of heuristic or greedy algorithms that are sensitive to the number of biological samples in each of the two sets of microarray data, as well as noise in the expression data. Therefore, it becomes unclear how the differences in the two networks will identify gene interactions that are linked to disease. Another approach [8] consists of incorporating an extra "cancer node" to the network in addition to the "gene nodes." This approach may yield a selection of genes related to cancer, but the mutual interrelationships of those genes with respect to cancer will not be revealed from the resulting network. Instead, we wish to introduce a novel type of graph with edges connecting pairs of genes that interact *with respect to cancer*, without including a cancer node. Phrased differently, the missing "cancer node" is associated with each connected gene pair (as opposed to individual genes) in the *whole *graph, so that the edges of the graph identify the gene pairs that are cooperatively associated with cancer. This kind of three-way representation is not feasible in a graph whose nodes are genes augmented by a cancer node. Thus, this methodology provides insight that existing methods cannot provide.

Recently, microarray data have been extensively analyzed at the level of gene modules, rather than individual genes, using prior biological knowledge [9-14], thus facilitating a higher-level view of the effects of diseases on gene expression. In contrast, our technique operates at the level of gene pairs and does not make use of prior biological knowledge.

What is a proper quantitative criterion to determine if two genes "interact with respect to cancer"? We could consider some measure of correlation between their joint expression levels and cancer. However, while this approach is proper if used for *classification *based on gene pairs, it is not appropriate for our purposes, because this correlation may be due to the independent contributions of the individual correlations between each of these genes and cancer, in which case the two genes do not interact. It is important to ensure that the correlation of a gene pair with cancer is due to *cooperative *effects, as opposed to independent contributions of the individual correlations. Such cooperative effects suggest a functional significance. Examples out of many possible biological reasons are the joint presence of two transcription factors each of which has a binding site in a promoter of an oncogene; the joint presence of a kinase and a transcription factor that must be activated; and the joint presence of the two elements of a dimeric transcription factor. In each of these cases, the two corresponding genes are strongly associated with cancer jointly, but not as much individually. On the other hand, individual oncogenes may not appear in our resulting graph, unless they are accompanied by properly identified "partner genes" to which they link. Traditional gene interaction network inference algorithms may then work in a complementary fashion to help identify interactions of the oncogenes that may not cooperative with respect to cancer.

To address this problem, we use the information theoretic measure of synergy [15]. The synergy of a gene pair with respect to cancer is defined as *I*(*G*_1_, *G*_2_; *C*) - [*I*(*G*_1_; *C*) + *I*(*G*_2_; *C*)], where *I *is the symbol for mutual information [16], *G*_1 _and *G*_2 _are random variables representing the expression levels of the two genes and *C *is a binary random variable representing the presence or absence of cancer. It can be seen as the "whole" minus the "sum of the parts." Intuitively speaking, if the amount of information that a pair of genes jointly provides about cancer is higher than what could be attributed to the additive independent contributions of the two individual genes, then this suggests that the additional information is due to some cooperative (direct or indirect) interaction involving these genes within a shared pathway. This is consistent with the definition of the word "synergy" (American Heritage Dictionary) as "the *interaction *of two or more agents or forces so that their combined effect is greater than the sum of their individual effects."

We define two genes to be "synergistically linked with respect to a phenotype" if their corresponding synergy is positive. These links can be depicted as edges in a graph representing a "**synergy network**," in which nodes are genes, depicting potential gene-gene interactions associated with a phenotype. In this paper, we include in the synergy network those edges corresponding to statistically significant synergies. When coupled with biological knowledge, this graph provides clues helpful for deciphering pathways responsible for the phenotype.

In previous work [15,17,18] these quantities were defined only for bilevel gene expression data, i.e., assuming genes are either "on" or "off," using arbitrary thresholds to binarize expression values inferred from microarrays. Here we introduce a novel dendrogram-based computational methodology generalizing these definitions by applying them directly to the continuous expression levels, allowing identification of high-synergy gene pairs. We apply this methodology on publicly available prostate cancer microarray data [19]. One of our main conclusions from the analysis of these data is that *RBP1 *(cellular retinol-binding protein-1, also known as *CRBP-1*) is synergistically linked with respect to prostate cancer with many other "partner" genes, many of which are ribosomal genes. Our results are also supportive of the hypothesis that prostate cancer is linked with cellular damage from oxidative stress combined with the inhibition of the apoptotic mechanisms normally resulting from such damage.

## Results

### Illustrating examples

**Figure 1:**
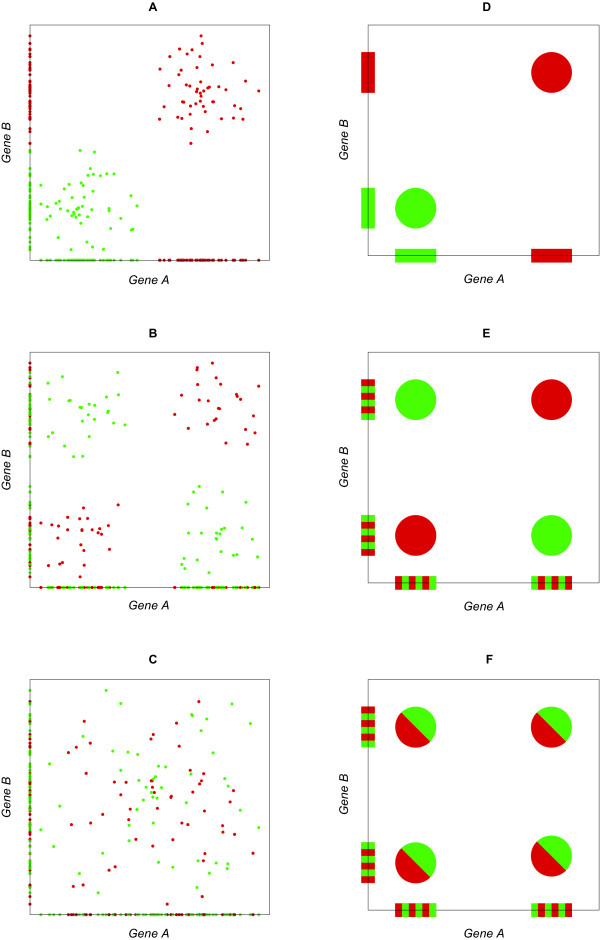
**Examples of scatter plots from the gene expression levels of two hypothetical genes illustrating the concept of synergy**. There is an equal number of red and green dots representing cancerous and healthy samples, respectively, therefore *H*(*C*) = 1 and the synergy is bounded by -1 and +1, where *C *is the symbol for the presence of cancer (see Methods). Also shown are the projections of the scatter plots to each of the two axes, thus allowing visualization of the relationship of each gene to cancer. Panels d, e, f show scatter plots of binarized expression levels corresponding to panels a, b, c, respectively, depicting circles for the multiple point at the vertices. (A) Each of the two genes ("oncogenes" in this case) is by itself sufficient to determine the presence or absence of cancer and there is negative synergy (redundancy). (B) In combination, the two genes are sufficient to determine the presence or absence of cancer, but each of them individually is uncorrelated with cancer and the synergy is positive. (C) Both genes, including their combination, are uncorrelated with cancer, and the synergy is approximately zero. (D) Illustration for the scatter plot from the binarized expression levels in panel a. The synergy is equal to -1. (E) Illustration for the scatter plot from the binarized expression levels of panel b. The synergy is equal to +1. (f) Illustration for the scatter plot from the binarized expression levels of panel c. Split bi-colored circles indicate the simultaneous presence of an equal number of healthy and cancerous samples. The synergy is equal to 0.

To clarify how the synergy can be measured from a set of continuous expression values, we consider some hypothetical extreme examples of two-dimensional gene expression scatter plots (Figure 1). The expression level of each of two "oncogenes" (Figure 1A) is sufficient by itself to distinguish health from cancer. On the other hand, the expression level of each of two genes may be totally uncorrelated with cancer (and therefore these genes would not be present in the output of any "gene ranking" computational method), and yet the pair of these two expression levels is also sufficient to distinguish health from cancer (Figure 1B), because cancer occurs when the two genes are either both "on" or both "off." It is also possible that the expression levels of two genes are totally uncorrelated with cancer, and so is the pair of the two (Figure 1C).

The amount of information that the expression level(s) of one or more genes provide about cancer can be quantified from the set of gene expression data using information theoretic tools [16]. For example, if *G *designates the expression levels of a gene and *C *designates the presence or absence of cancer, then the uncertainty of cancer given these two expression levels is equal [17] to the conditional entropy *H*(*C*|*G*), and the amount of information that the gene provides about cancer is equal to the mutual information *I*(*G*; *C*). These quantities are directly generalized if we replace the expression *G *of a single gene with the set of expression levels of all members of a gene set. The amount of information about cancer that is due to the *purely cooperative *effects among all the members of a gene set can also be quantified using information theoretic tools [15,18], specifically the synergy of a gene pair with respect to cancer previously defined as *I*(*G*_1_, *G*_2_; *C*) - [*I*(*G*_1_; *C*) + *I*(*G*_2_; *C*)]. It is possible for synergy to be negative (redundancy), as well as positive. Intuitively, we see that the synergy of the hypothetical genes in Figure 1A is negative, because of the underlying redundancy (each gene is sufficient by itself to determine if there is cancer), while the synergy of the genes in Figure 1B is positive, because the combination of the two genes is required for such determination.

If we are interested in classification of cancer based on a gene pair, then we wish to select the two genes that minimize the conditional entropy *H*(*C*|*G*_1_, *G*_2_), or, equivalently, maximize the mutual information *I*(*G*_1_, *G*_2_; *C*). If, however, we wish to infer biomolecular interactions related to cancer, then the "figure of merit" should be the synergy *I*(*G*_1_, *G*_2_; *C*) - [*I*(*G*_1_; *C*) + *I*(*G*_2_; *C*)]. These are two different tasks, and in this paper we focus on the latter.

### Evaluation of synergy from expression data

If the expression levels have been binarized so that each of the genes is in one of two expression states (0: "off" and 1: "on"), then the evaluation of the uncertainty in the form of conditional entropy is straightforward [17]. For example, each pair of genes has only four expression states (00, 01, 10, 11), and we can collect statistics by counting how many times each of these four states is encountered in health and in disease. Information theoretic quantities such as entropy and mutual information can then be evaluated from the probabilistic model that results from the relative frequencies (see Methods). Figures 1D, 1E, 1F illustrate the concept of binarized expression data from the corresponding scatter plots of Figures 1A, 1B, 1C, respectively. The resulting synergies can easily be evaluated as -1, +1, 0, respectively.

Binarization of expression data imposes a constraint that limits the usefulness of these techniques. Each gene has its own optimum "binarization threshold" to distinguish when it is "on" or "off," which is not clearly defined, and even if it was, significant information will still be lost by not accounting for the precise intermediate expression levels. We addressed these shortcomings by generalizing the above methodology to directly obtain measures of entropy, mutual information, and synergy from continuous gene expression data without any binarization, as explained below.

It is intuitively clear that the uncertainty will be low if the joint expression levels can be partitioned into clusters of samples with similar joint expression levels, so that each of the resulting clusters is "homogeneous," i.e., it contains predominantly healthy or predominantly diseased samples. This is the case in the two-dimensional scatter plots in Figure 1A and Figure 1B (but not in Figure 1C) as well as the one-dimensional projections for each of the two genes in Figure 1A (but not in Figure 1B and Figure 1C).

**Figure 2:**
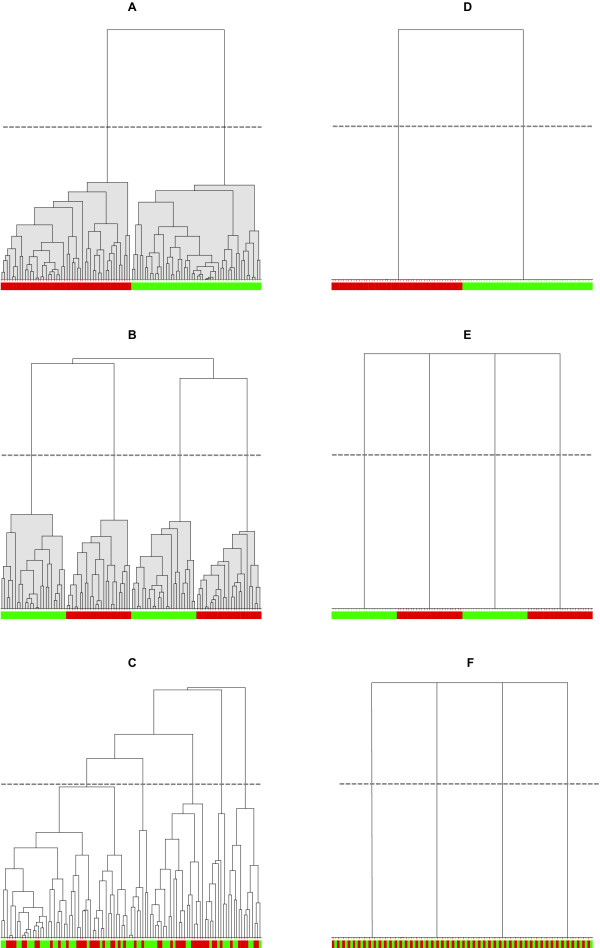
**Dendrograms for the two-dimensional scatter plots of Figure 1**. There is one-to-one correspondence between the six panels (A-F) of the two figures. Similar dendrograms for the one-dimensional (individual genes) projections of the scatter plots (not shown) are also needed for the evaluation of synergy. The leaves represent samples color-coded as red for cancer and green for health. For each dendrogram, a horizontal line defines a partition into clusters. In A and B there is perfect classification via partition into two and four homogeneous clusters, respectively. In c there is random assignment of samples resulting in inhomogeneity in each cluster and inability to classify. In D, E, F, the outcomes are the same as in A, B, C, respectively, except that the edges of the dendrogram fully connect the root with the leaves and any distance of the horizontal from the leaves will produce the same partition.

If the expression data are binarized, then each joint expression state automatically defines a "cluster" of co-located samples, as in Figures 1D, 1E and 1F, in which case evaluation of all information theoretic quantities are straightforward, as explained earlier. For example, the average uncertainty of predicting whether or not a sample is cancerous is equal to the average entropy of the joint expression states [15,17,18]. Our proposed computational methodology that directly uses continuous expression values generalizes this concept in a consistent ("backwards compatible") manner, because the average uncertainty of cluster-classifying whether or not a sample is cancerous is equal to the average entropy of the clusters partitioning the set of joint gene expression data (see Methods). Figure 2 shows dendrograms resulting from clustering for the corresponding cases in Figure 1. For each dendrogram, a horizontal line whose distance from the leaves of the tree is meant to represent a threshold of biological significance, defines a partition of the samples into a number of clusters, each of which has associated binary entropy related to the homogeneity of its class labels. The average of these individual cluster entropies, weighted by the relative membership of each cluster, defines the conditional entropy of cancer given the choice of gene(s), out of which measures of mutual information and synergy can directly be evaluated (see Methods).

In summary, we first introduce a methodology for estimating the uncertainty (conditional entropy) of predicting whether a sample is affected by a disease given the continuous expression levels of few particular genes in a number of diseased and healthy samples. For example, estimation from a number of samples of the conditional entropy *H*(*C*|*G*_1_, *G*_2_) where *C *is a binary random variable and *G*_1_, *G*_2 _are continuous random variables is a problem that has not been addressed before. The synergy between two genes can then be directly evaluated using this measure.

To confirm the validity of our methodology we applied our results on a simulated expression data set (Additional File 1) in which several gene pairs were assumed to be jointly associated with cancer (traditional synthetic network methods in which genes are assumed to be regulated by other genes are not applicable in this context). Our results confirm that the synergy methodology accurately deciphers these associations. For comparison, we also apply a traditional gene interaction inference method in various ways on these data to illustrate why such methods cannot be used to infer interaction *with respect to cancer*, as explained earlier.

### Application to prostate cancer gene expression dataset

We did an exhaustive search through all gene pairs in a publicly available prostate cancer data set, identifying the gene pairs with highest synergy and validated our results by confirming that their *P *values are extremely small and hypothesized on their biological interpretation. We applied our methodology on publicly available prostate cancer expression data [19] from 102 prostate samples, 50 of which were deemed to be healthy and 52 of them cancerous using RMA-normalized values (see Methods).

We first ranked all genes in terms of their conditional entropy *H*(*C*|*G*_*i*_). The ten lowest scoring genes are shown in Table 1. These are the genes that are individually most correlated with cancer, because the same genes would equivalently have been found as highest scoring in terms of the mutual information *I*(*G*_*i*_; *C*). Nearly all genes in the list are well-known biomarkers of prostate cancer, such as *HPN *[20]*ERG*[21], *AMACR *[22], *FOLH1 *[23], *TACSTD1 *[24] and *AGR2 *[25], thus validating the dendrogram-based technique for estimating entropy.

**Table 1:** Ranking of individual genes by entropy

	Symbol	Accession Number	Entropy
1	*HPN*	X07732	0.5151
2	*TRGV3*	M30894	0.6164
3	*PDLIM5*	AL049969	0.6503
4	*ERG*	M21535	0.6640
5	*AMACR*	AJ130733	0.6809
6	*NELL2*	D83018	0.6838
7	*CFD*	M84526	0.6917
8	*FOLH1*	M99487	0.6969
9	*TACSTD1*	M93036	0.6973
10	*AGR2*	AF038451	0.7090

**Figure 3:**
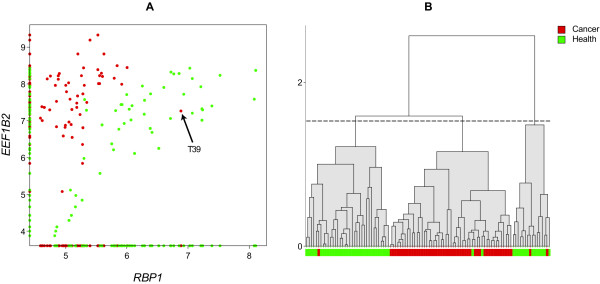
**Highest-synergy pair of genes**. Genes *RBP1 *and *EEF1B2 *in combination appear to predict prostate cancer in ways that cannot be attributed to the additive individual contributions of the genes (see *P *values in Table 2). (A) Scatter plot for the two genes. Red and green dots represent 52 cancerous and 50 healthy samples, respectively. The red dot indicated by the arrow represents sample T39, which appears to have been mislabelled as cancerous, a possibility also supported by scatter plots involving totally different genes (Figure 6). (B) Dendrogram for the corresponding two-dimensional scatter plot indicating a partition with good classification performance.

Using exhaustive search, we then also ranked all gene pairs in terms of their synergy *I*(*G*_*i*_, *G*_*j*_; *C*) - [*I*(*G*_*i*_; *C*) + *I*(*G*_*j*_; *C*)] (the 20 highest scoring pairs are shown in Table 2). The *P *value entries are explained in Methods. Notably, the top-ranked genes in individual gene ranking (Table 1) are different from those in the highest-synergy gene pairs (Table 2), consistent with the expectation that pairs of synergistically linked genes with respect to cancer are not necessarily individual cancer biomarkers. Figure 3 shows the corresponding scatter plot and dendrogram of the highest-synergy gene pair (*RBP1 *and *EEF1B2*).

**Table 2:** Ranking of gene pairs by synergy

	Symbol 1	Accession 1	Symbol 2	Accession 2	Synergy	*P *value
1	*RBP1*	M11433	*EEF1B2*	X60489	0.4025	< 1E-15
2	*RBP1*	M11433	*FTL*	M10119	0.3653	< 1E-15
3	*RBP1*	M11433	*HLA-DPB1*	M83664	0.3493	< 1E-15
4	*PTGDS*	M98539	*YWHAQ*	X56468	0.3408	< 1E-15
5	*RBP1*	M11433	*UQCRH*	Y00764	0.3348	< 1E-15
6	*RBP1*	M11433	*UBC*	AB009010	0.3331	< 1E-15
7	*RBP1*	M11433	*SNRPB*	AL049650	0.3287	< 1E-15
8	*RBP1*	M11433	*ZNF146*	AJ011806	0.3271	< 1E-15
9	*RBP1*	M11433	*EEF1D*	Z21507	0.3239	< 1E-15
10	*PTGDS*	M98539	*SLC25A6*	J03592	0.3202	4.00E-15
11	*RBP1*	M11433	*SLC25A6*	J03592	0.3202	5.00E-15
12	*RBP1*	M11433	*RPS15*	J02984	0.3199	7.00E-15
13	*RBP1*	M11433	*RPL5*	U14966	0.3177	9.60E-14
14	*RBP1*	M11433	*HLA-DRB5*	M32578	0.3169	2.47E-13
15	*RBP1*	M11433	*KPNA4*	AB002533	0.3138	6.97E-12
16	*RBP1*	M11433	*GAPDH*	M33197	0.3138	7.30E-12
17	*RBP1*	M11433	*MCL1*	L08246	0.3137	7.60E-12
18	*RBP1*	M11433	*RPS19*	M81757	0.3133	1.10E-11
19	*RBP1*	M11433	*PCBP2*	X78136	0.3090	5.94E-10
20	*RBP1*	M11433	*NCL*	M60858	0.3081	1.25E-09

**Figure 4:**
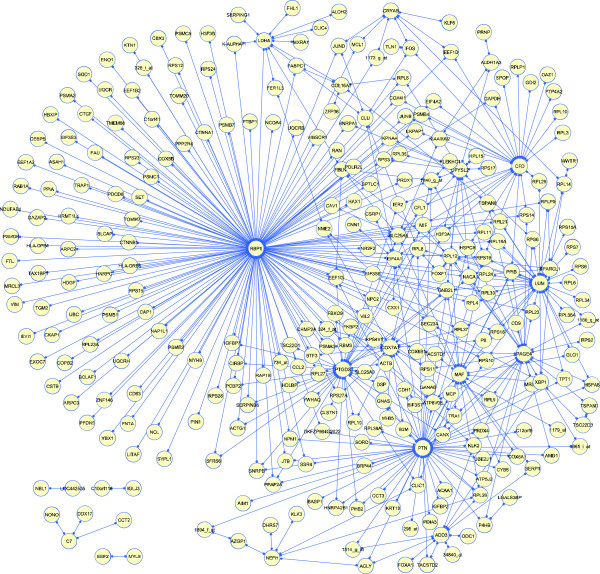
**Synergy network**. The network can be seen as a first effort to depict the "prostate cancer interactome" based on the analyzed expression dataset that included 50 healthy and 52 cancerous samples. Each edge depicts inferred gene-gene interactions associated with prostate cancer. Each node of the graph represents a gene. Gene pairs whose synergy has *P *< 0.05 under permutation B (see text) are indicated by the edges of the graph.

To determine the extent to which our numerical results could be due to pure chance we performed statistical validation experiments by repeating the identical computational procedures after permuting the gene expression matrix (see Methods). The resulting synergy network and a listing of the top-ranked gene pairs with their *P *values are shown in Figure 4 and Table 2, respectively.

### Validation with independent gene expression dataset

To confirm that our results are applicable when used on independently obtained samples, we used a prostate cancer gene expression dataset containing values for 25 malignant and 8 healthy samples from a different laboratory [26], to which we refer as the "validation dataset." We found that direct numerical evaluation of synergy from the validation dataset is not meaningful, because the *P *value for even the top-ranked gene pair is 0.10 (Additional File 2), indicating that results are not statistically significant.

**Figure 5:**
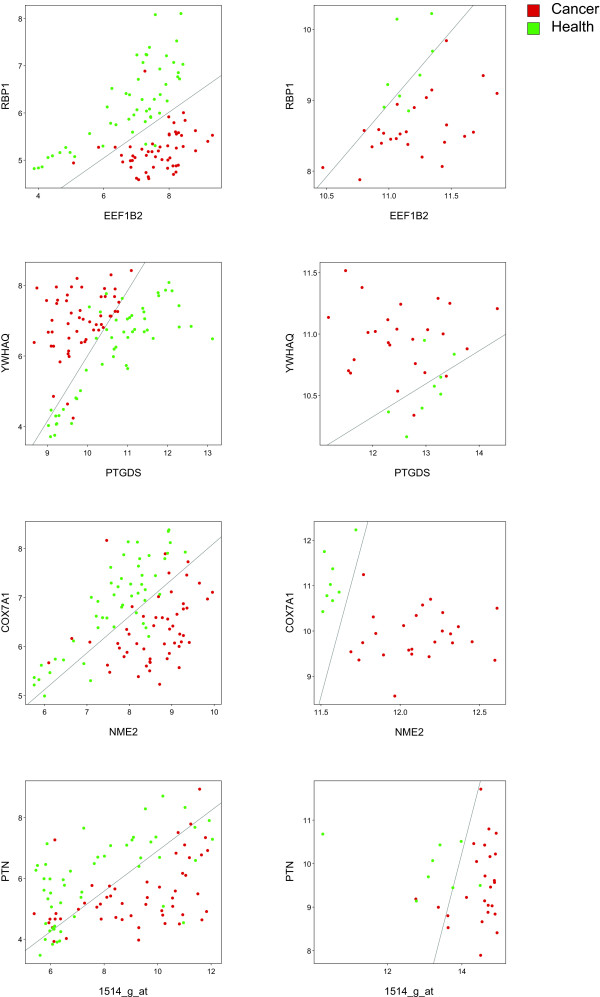
**Scatter plots of top-ranked gene pairs invalidation data set**. On the left is the original data set, and on the right is the validation data set of the top-ranked gene pairs, restricted to those with unique members, in terms of synergy. A separating line was computed for each plot using a Support Vector Machine with a linear kernel and an error penalty parameter of 10^6^.

In our case, the figure of merit, synergy, is not measurable by any classification performance. Rather, the high synergy of a gene pair with respect to a phenotype is due to a Boolean logic connection between the gene pair and the phenotype, such as "prostate cancer tends to occur only in the simultaneous expression of gene A and lack of expression of geneB." Our approach is aimed at deriving such logic relationships, as they are the ones that may lead to valuable biological insights. Therefore, a qualitative validation should focus on those logic relationships. Figure 5 shows the scatter plots of the top-ranked gene pairs in both the original and the validation dataset, chosen so that each new gene pair does not contain any gene previously used for this purpose (because the scatter plots tend to be the same in that case). For better illustration of the concept, we also include for each scatter plot the separating line derived from a linear Support Vector Machine with an error penalty parameter of 10^6^. It is clear that the tendency for the location of the joint expression levels is preserved in all cases.

## Discussion

**Figure 6:**
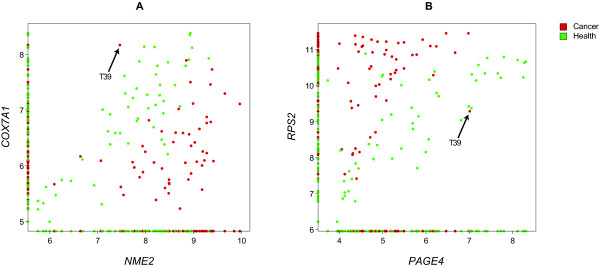
**Illustration of consistency among synergistic pairs**. The same sample (T39) consistently appears mislabelled, as was the case in Figure 3A, as cancerous on multiple scatter plots involving different genes. Shown are two additional examples, out of many, thus supporting the relevance of our results. All shown gene pairs in the scatter plots also appear in the pairwise synergy graph (Figure 4).

The purpose of this paper is twofold. First, to disclose a novel methodology of analyzing continuous microarray data aimed at discovering sets of genes synergistically associated with a phenotype such as cancer. Second, to apply this methodology on a set of expression data identifying gene pairs whose high values of synergy cannot be explained by pure chance, suggesting biological significance. These tasks have been achieved, as evidenced by the extremely low *P *values (Table 2) corresponding to some of the gene pairs. As an additional indication of the biological relevance of our results, we present two examples of scatter plots (Figure 6), in which the same sample (T39) consistently appears to be mislabelled as cancerous, as was the case in Figure 3A.

The next step would be to interpret the results aiming to better understand mechanisms responsible for prostate cancer and this requires coupling with existing biological knowledge. For example, although we may have established the synergistic association of a gene pair with respect to prostate cancer, this does not necessarily mean that the interaction of these two genes causes the cancer; it is, however, an indication that these two genes interact, directly or indirectly, within a shared pathway that is associated with cancer, but the cause-versus-effect relationship between them is unclear. Here, we mention some already known facts involving the genes in the highest-ranking pairs. We hope that current and future biological knowledge will lead to a satisfactory biological interpretation of these results, helpful for inferring biological mechanisms responsible for prostate cancer.

The gene that appears in most of the high-synergy gene pairs is *RBP1 *(cellular retinol-binding protein-1). It turns out that all the gene pairs in Table 2 that include *RBP1 *are governed by the same molecular logic: prostate cancer tends to occur only when the following two events occur simultaneously: (a) *RBP1 *is expressed at low levels and (b) its partner gene is expressed at high levels. It is known that *RBP1 *is downregulated in several cancers, and an explanation has been proposed based on the fact that *RBP1 *inhibits the PI3K/Akt survival pathway [27], therefore downregulation of *RBP1 *promotes derepression of PI3K/Akt signalling and inhibits apoptosis of malignant cells. It has also been found that hypermethylation of *RBP1 *is common in several tumors and cancer cell lines [28], which may partly explain its downregulation.

Another central gene with multiple partner genes (Figure 4) is *PTGDS *(prostaglandin D_2 _synthase, also known as *L-PGDS*). Interestingly, *PTGDS *has also been linked to apoptosis concomitant with downregulation of PI3K [29]. We found that a synergistic gene pair that includes *PTGDS *obeys the same "molecular logic" as *RBP1*, i.e. prostate cancer occurs in the simultaneous low expression of *PTGDS *and high expression of the partner gene.

There are several synergistic "partner genes" for *RBP1 *or *PTGDS *some of which (such as *SLC25A6*) serve as partner genes to both (Table 2). The top-ranked gene pair consists of *RBP1 *and *EEF1B2 *(eukaryotic elongation factor 1-beta, also referred to as *eEF1Bα*).

Many of the highest-ranking synergistic partner genes for *RBP1 *and *PTGDS *(Table 2) are associated, directly or indirectly, with oxidative stress, including *EEF1B2 *[30]; *FTL *[31] (ferritin, light polypeptide); *HLA-DPB1 *[32] (major histocompatibility complex, class II, DP beta 1), *YWHAQ *[33] (tyrosine 3-monooxygenase/tryptophan 5-monooxygenase activation protein, theta polypeptide- also known as 14-3-3), *UQCRH *(ubiquinol-cytochrome c reductase hinge protein), known to be involved in oxidative phosphorylation, and *UBC *[34] (UbiquitinC). Furthermore, *SLC25A6 *(solute carrier family 25 – mitochondrial carrier; adenine nucleotide translocator – member 6) is also known to be involved in oxidative phosphorylation.

Taken together the above observations are consistent with the previous [17] speculation that these microarray data indicate that prostate cancer is often associated with cellular damage caused by oxidative stress combined with inhibition of the apoptotic mechanisms that are normally triggered by the damage. They are also consistent with other recent results [35] linking prostate cancer with oxidative stress.

Another observation is that many of the genes serving as synergistic partners to *RBP1 *(Figure 4) are ribosomal, including genes directly encoding for ribosomal proteins as well as translation elongation and initiation factors. The mRNAs of most such genes share a 5'-terminal oligopyrimidine tract (TOP) used for translational control. It has been proposed that oncogenic transformation by Akt involves intervention in translational controls through the mTOR pathway, which activates the kinase S6K and controls the translation of 5'TOP mRNAs [36,37]. The mRNAs of ribosomal genes including elongation and initiation factors are often up-regulated in expression profiles from diverse tumors and clustered together [38]. Furthermore, the variation in expression of these ribosomal genes was found significantly correlated with variation in the cell doubling time, supporting the notion that the genes in this cluster were regulated in relation to cell proliferation rate or growth rate [38].

The high synergy between *RBP1 *and the ribosomal genes is largely reflected by the fact that there are a number of samples that are healthy despite the fact that *RBP1 *is underexpressed. These are the samples for which the ribosomal genes are underexpressed, for example those corresponding to the lowest nine green dots in the *RBP1/EEF1B2 *scatter plot (Figure 3A). We speculate that mRNA underexpression of the cluster of ribosomal genes protects the organism from cancer by inhibiting cell proliferation even if the cell has been damaged, apoptosis is inhibited and the Akt survival pathway is activated as a result of *RBP1 *underexpression.

In addition to *RBP1 *and *PTGDS*, several other genes appear in central positions in Figure 4, some of which are already known to participate in pathways jointly involving prostate cancer and oxidative stress, such as *PTN *(also known as *HARP*) [39] and *CLU *[40]. Knowledge of their "interacting partner" genes, as provided by the synergy network, can help identify the precise nature of these pathways.

## Conclusion

The unique feature and strength of using synergy networks resulting from gene expression analysis is that it focuses on finding genes that are *cooperatively *correlated with disease, rather than just correlated with disease, and therefore it can be helpful for the inference of pathways responsible for disease. Furthermore, identification of gene pairs that are synergistically associated with disease has obvious applications in combinatorial approaches for treatment, as single targets would, but the very definition of synergy, not be sufficient. In this paper we have introduced a methodology estimating synergy directly from continuous expression data for tens of thousands of genes, with sufficiently low computational complexity allowing exhaustive search of all gene pairs (see Methods). Our technique is also extendable to include any types of biomarkers, including alternatively spliced isoforms and protein expression or post-translation modification data, shedding further light on putative responsible pathways.

Synergy networks are *complementary *to, and different from, traditional gene interaction networks. The success of our results cannot be measured by prediction accuracy, because the aim is not classification. Instead, we seek to find gene pairs whose combined information correlates with a phenotype better than the sum of either gene individually. Furthermore, traditional gene interaction algorithms operate without any reference to cancer, and they can be useful for identifying interactions involving known oncogenes, which by themselves are unrelated to cancer and our technique would not identify. Each approach provides valuable information that the other cannot provide.

Our proposed methodology requires access to a set of gene expression data that is rich not only for diseased but also for currently rare healthy (control) samples to ensure the statistical significance of the results. Using such rich and balanced datasets, the same methodology can be generalized to discover synergistic triplets of genes using the definition of multivariate synergy [15], which will provide additional and more significant information helpful for pathway inference, as it will suggest that all three genes in the triplet will be members of a shared pathway. Such collection of high-quality standardized gene expression data is not expensive compared with other efforts such as sequencing, and we hope that it becomes incorporated in some of the existing or future cancer initiatives.

## Methods

### Entropy of a clustering partition

Given a cluster of samples, each of which is assigned one of two possible class labels referred to by the symbol *C*, in our case health (*C *= 0) versus a particular cancer (*C *= 1), we define the entropy of the cluster as *h*(*Q*) = -*Qlog*_2 _*Q *- (1-*Q*) *log*_2_(1-*Q*), where *Q *is the relative frequency of cancerous samples in the cluster. Given a partition of the full set of samples into a number of disjoint clusters, we define the entropy of the partition as the average of the entropies of all clusters, weighted by the relative membership of each cluster. For example, assume that there are totally *K*_0 _healthy samples and *K*_1 _cancerous samples with *K*_0 _+ *K*_1 _= *K *and that one of the clusters contains *N*_0 _healthy samples and *N*_1 _cancerous samples. It follows that the relative membership of the cluster is *P *= (*N*_0 _+ *N*_1_)/*K *and the entropy of the cluster is *h*(*Q*) where *Q *= *N*_1_/(*N*_0 _+ *N*_1_). Therefore, the entropy of the partition will be equal to the sum ∑*Ph*(*Q*) over all clusters.

### Conditional entropy

Assume that each choice of *n *genes defines a partition of the samples according to a clustering algorithm applied on the expression levels of these genes in all the samples. Given such a choice of genes with expression levels denoted by the symbols *G*_1_,...,*G*_*n*_, the conditional entropy of the class label *C *is equal to the entropy of the resulting partition, i.e., *H*(*C*|*G*_1_,...,*G*_*n*_) = ∑*Ph*(*Q*), and measures the average uncertainty of predicting if a sample is cancerous if we know the cluster in which the sample is located. In the special case that the expression levels *G*_*i *_are binary so that each gene is either "off" (*G*_*i *_= 0) or "on" (*G*_*i *_= 1), then this methodology becomes identical to evaluating the same conditional entropy from the probabilistic model resulting from relative frequencies after counting the number of healthy and cancerous samples in each of the 2^*n *^possible expression states [15,17,18].

### Mutual information

The mutual information *I*(*G*_1_,...,*G*_*n*_; *C*) is a nonnegative quantity measuring the information that the *n *genes provide about cancer and is equal to *H*(*C*) - *H*(*C*|*G*_1_,...,*G*_*n*_), where *H*(*C*) is equal to *h*(*K*_1_/*K*), in our case *K*_1 _= 50 and *K *= 102, so *H*(*C*) = 0.9997. We further normalized mutual information and conditional entropy by dividing by *H*(*C*) so that in their normalized form I*(*G*_1_,...,*G*_*n*_; *C*) = 1 - H*(*C*|*G*_1_,...,*G*_*n*_), so that the maximum normalized possible mutual information in the values of Table 1 is equal to one.

### Synergy

When *n *= 2, the synergy [15]*Syn*(*G*_1_, *G*_2_; *C*) measures the amount of information about cancer that is due to purely cooperative effects between *G*_1 _and *G*_2 _and is equal to *Syn*(*G*_1_, *G*_2_; *C*) = *I*(*G*_1_, *G*_2_; *C*) - [*I*(*G*_1_; *C*) + *I*(*G*_2_; *C*)], which is also equal to *H*(*C*|*G*_1_) + *H*(*C*|*G*_2_) - *H*(*C*|*G*_1_, *G*_2_) - *H*(*C*). We further normalized the synergy, as we did for the mutual information, by dividing by *H*(*C*), so that, in its normalized form, the maximum synergy in the values of Table 2 is equal to 1.

### Evaluation of conditional entropy

Given a choice of *n *genes we wish to numerically estimate the corresponding conditional entropy ∑*Ph*(*Q*), to which for simplicity we will refer in this paragraph using the symbol *H*, from the continuous expression levels of these genes. We used the UPGMA clustering algorithm [41] applied on the genes' RMA-normalized joint expression levels. Corresponding dendrograms can be plotted with the root at the top and the leaves in a horizontal line at height 0. Each horizontal line (Figure 3) at distance *D *from the leaves defines a partition into clusters for which a value *H *can be computed. The value of *H *will change discontinuously with *D *as pairs of clusters are merged into single clusters each time the horizontal line crosses the intermediate nodes of the dendrogram by moving higher. This discontinuity is undesirable, particularly because the formula for evaluating synergy involves three independent calculations of mutual information (one for the pair of genes and two for each gene alone) thus occasionally amplifying inaccuracies due to borderline effects at the discontinuity points. Furthermore, evaluating *H *at a specific value of *D *does not account for potentially interesting partitioning detail that may occur within the sub-clusters below the horizontal line at *D*. To remedy these problems, we used a measure of the conditional entropy that averages *H *by integrating it from 0 up to a cut-off value D* and dividing by D*. The value of D* can be considered to be a "threshold of biological significance," because clusters with inter-cluster distances above D* are not merged. We used a value of D* = 1.5 on the RMA-normalized data (which are already log-normalized). We found that, when using this averaged value of *H *as figure of merit to be minimized over the choices of gene sets, there is not much sensitivity on the choice of D* in terms of the relative comparison of values of entropy or synergy associated with gene sets. For example, comparing the top 100 most synergistic pairs for D* = 1.5 to the top 100 pairs for D* = 1.25 and D* = 1.75, we found that there were 83 pairs in common for D* = 1.5 and D* = 1.25, and 76 pairs in common for D* = 1.5 and D* = 1.75. Furthermore, regardless of the choice of D*, this measure is still backwards compatible with the evaluation of the conditional entropy in binary expression data [15,17,18], in which case *H *is independent of D*, as illustrated in Figures 1D, 1E, 1F. To further estimate the sensitivity to the choice of the parameter, we compared the top 100 pairs with the top 100 pairs for D* = 1.5 The results for D* = 1.0, 1.25, 1.75 and 2.0 were 62%, 83%, 76% and 54%, respectively. Therefore, there is a reasonably wide range of the values of D* yielding consistent results. This sensitivity should not be expected to be very wide, as the biological meaning of the parameter is meant to be the threshold of biological significance, so that each cluster is interpreted as a biological event.

### Distance measure

When calculating the UPGMA dendrograms, we use the Chebyshev distance measure (i.e., the maximum distance in any single dimension), because synergy evaluation requires that entropy values computed over different numbers of dimensions (genes) be included in the same formula. As dimensions are added, Chebyshev distances remain limited by the maximum distance between the expression levels of two genes and therefore we can conveniently use the same value of D* for all dimensions; in contrast Euclidean distances steadily increase as more dimensions are added, making comparisons to different dimensions problematic. From a biological viewpoint, this choice assumes that the "threshold of biological significance" in the joint gene expression space of a synergistic set of genes is the same as the threshold for individual member genes. In other words, if the joint expression of two genes is causing a phenotype exclusively as a result of their synergistic interaction, then it is sufficient for one of them to exceed the threshold of biological significance for the pair of genes to cease causing the phenotype. When using the above-defined numerical measure of conditional entropy and the Chebyshev distance measure, we always found in our results that *H*(*C*|*G*_1_, *G*_2_) ≤ *min*{*H*(*C*|*G*_1_), *H*(*C*|*G*_2_)}, consistent with information-theoretic facts [16]. This was not always the case when we used other distance measures, such as the Euclidean distance.

### DNA microarray data set and normalization

Raw probe data (CEL files) for a set [19] of Affymetrix Human Genome U95Av2 microarray assays were obtained from the Broad Institute's website. The set consists of 102 assays: 52 prostate tumor samples and 50 non-tumor prostate samples. The microarray chip had probe sets for 12,625 features, which were normalized and summarized using the *Robust Multi-array Average *(RMA) method [42] on perfect match probes only. The implementation of RMA used was from Bioconductor 1.8 using default settings.

### Statistical analysis of validation experiments

We implemented two types of permutation on the gene expression matrix, whose rows correspond to the genes and columns correspond to the samples with the first 50 columns containing the healthy samples and the remaining 52 columns containing the cancerous samples: Under "permutation A" the columns are randomly shuffled so that the class labels (health versus cancer) of the samples are permuted. Thus, the expression profiles of the samples become uncorrelated with the class label, while the integrity of the gene interrelationships in individual samples is retained. Under "permutation B" each gene's expression values are independently shuffled twice, once within the healthy samples and once within the cancerous samples, so that the individual genes' association with the class difference is retained (for example oncogenes remain "oncogenes"), but the integrity of the gene interrelationships in individual samples is destroyed. It is not clear which of the two types is preferable for our purposes, since our definition of synergy makes use of both marginal as well as joint correlations; therefore we performed both of them.

Our first aim was to obtain an estimate of the statistical significance of the highest-synergy pair of the actual expression data compared with the highest-synergy pairs resulting from the permutation experiments. For this purpose, we did 100 permutation experiments of each type, saving the corresponding 100 *highest *synergies after doing exhaustive search in each permutation experiment. Using the set of these 100 highest-synergy scores, we obtained the maximum likelihood estimates of the location parameter and the scale parameter of the Gumbel (type-I extreme value) distribution, resulting in a cumulative density function *F*. The Gumbel distribution [43] is the limiting distribution of the maximum of a large number of random observations from the same arbitrary distribution. The *P*value of the maximum synergy *x*_0 _found in the actual data (defined, in this case, as the estimated probability of obtaining maximum synergy at least as high as *x*_0 _by pure chance when the null model includes the *highest *synergy values among $N=\left(\begin{array}{c} 12,625\\ 2 \end{array}\right)$ = ~80 million gene pairs for *each *permutation experiment) was then evaluated as 1-*F*(*x*_0_). For our highest-synergy pair (*RBP1 *with *EEF1B2*) we found *P *< 10^-15 ^for both permutations A and B.

To examine the effectiveness of estimating the Gumbel distribution using 100 permutations, we ran the following simulation experiment. We used the estimated Gumbel parameters (0.02536117 and 0.2314731) to simulate sets of 100 random numbers. For each set, we estimated the Gumbel distribution parameters based on the random drawn values. The process was then repeated 5,000 times. We compared the cumulative distribution function (CDF) according to the true simulation setup and those according to the sample estimates based on only 100 random values, demonstrating that the tail probability from the estimated distribution was very close to the true value. Specifically, the estimated parameters from 100 values (randomly drawn from the Gumbel distribution) were unbiased and with reasonable precision: The means were 0.02536843 and 0.2314643, while the corresponding "standard errors" were 0.002286060 and 0.002688641. Therefore, 100 simulations are sufficient to estimate the distribution.

Our second aim was to define a cut-off threshold of statistical significance for the gene pairs to be included in the synergy network. In that case, we cannot make use of the Gumbel distribution, because it only applies on the highest values. Furthermore, due to the large number *N *of gene pairs, it is important to adjust for multiple comparisons. A widely used procedure to adjust individual tests' significance controls the false discovery rate (FDR) [44], which is the expected proportion of falsely rejected hypotheses among all rejected.

Using *K *= 10^8 ^synergy scores randomly sampled from the permuted data, we computed for each synergy score on a gene pair *P*values adjusted for FDR. Let *S*_*i *_be the synergy score of gene pair *i*, and $S_{k}^{*}$ be the synergy score for permuted sampled pair *k *(*k *= 1,...,*K*). For the FDR-adjusted *P *value, we first sorted the synergy scores, so that *S*_1 _≥ *S*_2 _≥...≥ *S*_*N*_. The raw *P *value was then estimated [45] as

$${\hat{P}}_{i}=\frac{1}{K}\#\left(S_{k}^{*}\geq S_{i}\right),\;k=1,\ldots ,K$$

and the FDR-adjusted *P *value is

$${\hat{P}}_{i}^{FDR}=\underset{j\geq i}{\min}\left(\frac{N}{j}{\hat{P}}_{j}\right).$$

One can then control the FDR at arbitrary level *α *by subjecting ${\hat{P}}^{FDR}$ to threshold *α*. Using *α *= 0.05, we found 2,719 significant gene pairs under permutation A and 473 significant gene pairs under permutation B. The synergy graph containing those 473 gene pairs is shown in Figure 4, while the 20 top-ranked gene pairs are listed in Table 2.

### Implementation and complexity

We implemented an algorithm that, using exhaustive search, simultaneously computes the UPGMA clustering and the conditional entropy for each individual gene, as well as for each gene pair, from which we evaluated the synergy of each gene pair. We then ranked gene pairs in terms of conditional entropy and synergy. The search space was partitioned and run on a 200-node computing cluster, and the running time of the entire process (processing ~80 million gene pairs) was approximately one hour.

### Availability

Software for evaluating entropy and synergy in MATLAB is available in Additional File 3.

## Authors' contributions

JW and DA developed the algorithms, performed their computational implementation on all datasets and drafted the manuscript. TZ and XW provided the statistical validation and wrote the corresponding part of the manuscript. All authors read and approved the final manuscript.

## Supplementary Material

Additional file 1 Example with simulated dataset. Comparison between using synergy networks and traditional network inference techniques on a simulated dataset. Click here for file

Additional file 2 Synergy values in validation dataset. Results of applying the synergy network algorithm on an independent dataset used for validation. Click here for file

Additional file 3 Software for evaluating entropy and synergy. MATLAB scripts are provided for evaluating conditional entropy and synergy from gene expression data and a corresponding phenotype indicator. Click here for file

## References

[B1] Bansal M, Belcastro V, Ambesi-Impiombato A, di Bernardo D (2007). How to infer gene networks from expression profiles. Mol Syst Biol.

[B2] Pearl J (1988). Probabilistic Reasoning in Intelligent Systems: Networks of Plausible Inference.

[B3] Friedman N (2004). Inferring cellular networks using probabilistic graphical models. Science.

[B4] Butte AJ, Kohane IS (2000). Mutual information relevance networks: functional genomic clustering using pairwise entropy measurements. Pac Symp Biocomput.

[B5] Basso K, Margolin AA, Stolovitzky G, Klein U, Dalla-Favera R, Califano A (2005). Reverse engineering of regulatory networks in human B cells. Nat Genet.

[B6] Kishino H, Waddell PJ (2000). Correspondence analysis of genes and tissue types and finding genetic links from microarray data. Genome Inform Ser Workshop Genome Inform.

[B7] Schafer J, Strimmer K (2005). An empirical Bayes approach to inferring large-scale gene association networks. Bioinformatics.

[B8] Gevaert O, De Smet F, Timmerman D, Moreau Y, De Moor B (2006). Predicting the prognosis of breast cancer by integrating clinical and microarray data with Bayesian networks. Bioinformatics.

[B9] Mootha VK, Lindgren CM, Eriksson KF, Subramanian A, Sihag S, Lehar J, Puigserver P, Carlsson E, Ridderstrale M, Laurila E (2003). PGC-1alpha-responsive genes involved in oxidative phosphorylation are coordinately downregulated in human diabetes. Nat Genet.

[B10] Segal E, Friedman N, Koller D, Regev A (2004). A module map showing conditional activity of expression modules in cancer. Nat Genet.

[B11] Rhodes DR, Kalyana-Sundaram S, Mahavisno V, Barrette TR, Ghosh D, Chinnaiyan AM (2005). Mining for regulatory programs in the cancer transcriptome. Nat Genet.

[B12] Rhodes DR, Chinnaiyan AM (2005). Integrative analysis of the cancer transcriptome. Nat Genet.

[B13] Subramanian A, Tamayo P, Mootha VK, Mukherjee S, Ebert BL, Gillette MA, Paulovich A, Pomeroy SL, Golub TR, Lander ES, Mesirov JP (2005). Gene set enrichment analysis: a knowledge-based approach for interpreting genome-wide expression profiles. Proc Natl Acad Sci USA.

[B14] Tomlins SA, Mehra R, Rhodes DR, Cao X, Wang L, Dhanasekaran SM, Kalyana-Sundaram S, Wei JT, Rubin MA, Pienta KJ (2007). Integrative molecular concept modeling of prostate cancer progression. Nat Genet.

[B15] Anastassiou D (2007). Computational analysis of the synergy among multiple interacting genes. Mol Syst Biol.

[B16] Cover TM, Thomas JA (2006). Elements of information theory.

[B17] Varadan V, Anastassiou D (2006). Inference of disease-related molecular logic from systems-based microarray analysis. PLoS Comput Biol.

[B18] Varadan V, Miller DM, Anastassiou D (2006). Computational inference of the molecular logic for synaptic connectivity in C. elegans. Bioinformatics.

[B19] Singh D, Febbo PG, Ross K, Jackson DG, Manola J, Ladd C, Tamayo P, Renshaw AA, D'Amico AV, Richie JP (2002). Gene expression correlates of clinical prostate cancer behavior. Cancer Cell.

[B20] Magee JA, Araki T, Patil S, Ehrig T, True L, Humphrey PA, Catalona WJ, Watson MA, Milbrandt J (2001). Expression profiling reveals hepsin overexpression in prostate cancer. Cancer Res.

[B21] Rostad K, Mannelqvist M, Halvorsen OJ, Oyan AM, Bo TH, Stordrange L, Olsen S, Haukaas SA, Lin B, Hood L (2007). ERG upregulation and related ETS transcription factors in prostate cancer. Int J Oncol.

[B22] Rubin MA, Zhou M, Dhanasekaran SM, Varambally S, Barrette TR, Sanda MG, Pienta KJ, Ghosh D, Chinnaiyan AM (2002). alpha-Methylacyl coenzyme A racemase as a tissue biomarker for prostate cancer. Jama.

[B23] Pinto JT, Suffoletto BP, Berzin TM, Qiao CH, Lin S, Tong WP, May F, Mukherjee B, Heston WD (1996). Prostate-specific membrane antigen: a novel folate hydrolase in human prostatic carcinoma cells. Clin Cancer Res.

[B24] Went P, Vasei M, Bubendorf L, Terracciano L, Tornillo L, Riede U, Kononen J, Simon R, Sauter G, Baeuerle PA (2006). Frequent high-level expression of the immunotherapeutic target Ep-CAM in colon, stomach, prostate and lung cancers. Br J Cancer.

[B25] Zhang JS, Gong A, Cheville JC, Smith DI, Young CY (2005). AGR2, an androgen-inducible secretory protein overexpressed in prostate cancer. Genes Chromosomes Cancer.

[B26] Welsh JB, Sapinoso LM, Su AI, Kern SG, Wang-Rodriguez J, Moskaluk CA, Frierson HF, Hampton GM (2001). Analysis of gene expression identifies candidate markers and pharmacological targets in prostate cancer. Cancer Res.

[B27] Farias EF, Marzan C, Mira-y-Lopez R (2005). Cellular retinol-binding protein-I inhibits PI3K/Akt signaling through a retinoic acid receptor-dependent mechanism that regulates p85–p110 heterodimerization. Oncogene.

[B28] Esteller M, Guo M, Moreno V, Peinado MA, Capella G, Galm O, Baylin SB, Herman JG (2002). Hypermethylation-associated Inactivation of the Cellular Retinol-Binding-Protein 1 Gene in Human Cancer. Cancer Res.

[B29] Ragolia L, Palaia T, Paric E, Maesaka JK (2003). Elevated L-PGDS activity contributes to PMA-induced apoptosis concomitant with downregulation of PI3-K. Am J Physiol Cell Physiol.

[B30] Olarewaju O, Ortiz PA, Chowdhury WQ, Chatterjee I, Kinzy TG (2004). The Translation Elongation Factor eEF1B plays a role in the oxidative stress response pathway. RNA Biol.

[B31] Orino K, Lehman L, Tsuji Y, Ayaki H, Torti SV, Torti FM (2001). Ferritin and the response to oxidative stress. Biochem J.

[B32] Grimm M, Spiecker M, De Caterina R, Shin WS, Liao JK (2002). Inhibition of major histocompatibility complex class II gene transcription by nitric oxide and antioxidants. J Biol Chem.

[B33] Pendergast AM (2005). Stress and death: breaking up the c-Abl/14-3-3 complex in apoptosis. Nat Cell Biol.

[B34] Fernandes R, Ramalho J, Pereira P (2006). Oxidative stress upregulates ubiquitin proteasome pathway in retinal endothelial cells. Mol Vis.

[B35] Ouyang X, DeWeese TL, Nelson WG, Abate-Shen C (2005). Loss-of-function of Nkx3.1 promotes increased oxidative damage in prostate carcinogenesis. Cancer Res.

[B36] Aoki M, Blazek E, Vogt PK (2001). A role of the kinase mTOR in cellular transformation induced by the oncoproteins P3k and Akt. Proc Natl Acad Sci USA.

[B37] Wendel HG, De Stanchina E, Fridman JS, Malina A, Ray S, Kogan S, Cordon-Cardo C, Pelletier J, Lowe SW (2004). Survival signalling by Akt and eIF4E in oncogenesis and cancer therapy. Nature.

[B38] Ross DT, Scherf U, Eisen MB, Perou CM, Rees C, Spellman P, Iyer V, Jeffrey SS, Van de Rijn M, Waltham M (2000). Systematic variation in gene expression patterns in human cancer cell lines. Nat Genet.

[B39] Polytarchou C, Hatziapostolou M, Papadimitriou E (2005). Hydrogen peroxide stimulates proliferation and migration of human prostate cancer cells through activation of activator protein-1 and up-regulation of the heparin affin regulatory peptide gene. J Biol Chem.

[B40] Miyake H, Hara I, Gleave ME, Eto H (2004). Protection of androgen-dependent human prostate cancer cells from oxidative stress-induced DNA damage by overexpression of clusterin and its modulation by androgen. Prostate.

[B41] Sneath PHA, Sokal RR (1973). Numerical taxonomy; the principles and practice of numerical classification.

[B42] Irizarry RA, Hobbs B, Collin F, Beazer-Barclay YD, Antonellis KJ, Scherf U, Speed TP (2003). Exploration, normalization, and summaries of high density oligonucleotide array probe level data. Biostatistics.

[B43] Gumbel EJ (1958). Statistical-Theory of Extreme Values. Bulletin of the International Statistical Institute.

[B44] Benjamini Y, Hochberg Y (1995). Controlling the False Discovery Rate – a Practical and Powerful Approach to Multiple Testing. Journal of the Royal Statistical Society Series B-Methodological.

[B45] Yekutieli D, Benjamini Y (1999). Resampling-based false discovery rate controlling multiple test procedures for correlated test statistics. Journal of Statistical Planning and Inference.

