# Supplementary material for: Identification of gene interactions associated with disease from gene expression data using synergy networks
Source: BMC Syst Biol. 2008 Jan 30;2:10. doi: 10.1186/1752-0509-2-10 (PMC2258206; doi:10.1186/1752-0509-2-10)
Supplement: Additional file 1 — Example with simulated dataset. Comparison between using synergy networks and traditional network inference techniques on a simulated dataset. [file 1752-0509-2-10-S1.pdf]

Additional file 1

Example with simulated dataset

A simple simulated data set was generated to demonstrate the types of interactions discovered by the synergy method. The data set consists of expression values for 12 genes over 200 samples evenly split between *health* and *cancer*. The gene pair (G_1, G_2) has an "OR" relationship, in the sense that cancerous samples express at least one of these two genes, while healthy samples express neither of the two. The gene pairs (G_3, G_4), (G_3, G_5), (G_3, G_6) have an "AND" relationship, in the sense that cancerous samples express all these genes, while healthy samples express at most one in each of these three pairs. The remaining six genes are simple oncogenes or tumor suppressors; that is, their expression is correlated or anti-correlated with the cancer phenotype, respectively. Each value was drawn from a Gaussian distribution with a standard deviation 0.3 and a mean of 1 for "expressed" and -1 for "not expressed." Furthermore, a 5% error was introduced for each sample and each relationship. For example, in the computation of the expression values of an oncogene for each sample, there is a 95% chance of having the "correct" expression value and a 5% chance of having the "wrong" expression value. For gene pairs obeying an AND or OR logic, the probabilities of the four possible expression states were arranged so that the probability of the logic state referred by the logic function was either 95% or 5%, while the remaining three states were assumed equally likely.

Table A1-1 shows the "gene ranking" based on minimizing the entropy of cancer for each single gene. As expected, the oncogenes and tumor suppressors, and not the members of the synergistic genes, were at the top of that list.

Table A1-2 shows the most synergistic pairs of genes with respect to cancer. As expected, the pairs achieving the top four synergy scores were (G_3, G_5), (G_1, G_2), (G_3, G_6), (G_3, G_4). Indeed, these were the only pairs that achieved positive synergy scores.

Finally, Table A1-3 shows the top-ranked pairs of genes based on minimizing the entropy of cancer for each pair of genes. In that case, pairs of independently contributing oncogenes are found at the top of the list, although synergistic gene pairs also have low entropy. This demonstrates that if two genes are jointly high correlated with cancer, this does not necessarily imply a cooperative relationship with respect to cancer. Indeed, the gene pairs at the top of the list have negative synergy (redundancy), indicating that their strong association to cancer is due to independent, rather than cooperative, effects.

For comparison, we also applied a gene interaction network construction method to this simulated dataset for the healthy samples (Figure A1-1), the cancerous samples (Figure A1-2), all samples (Figure A1-3) and all samples including an additional "cancer outcome node" (Figure A1-4). The networks were constructed using the GeneNet R package for graphical Gaussian models downloaded from

http://strimmerlab.org/software/genenet/index.html [1]. Default parameters and methods were used according to the documentation of the GeneNet authors.

These figures clarify the fact that networks resulting from traditional gene-gene interaction inference are designed to infer interactions among member genes and therefore, but their own nature, do not directly capture synergistic genes pairs with respect to cancer. If a cancer node is included, it tends to form links with the individual oncogenes.

References

1. Schafer J, Strimmer K: **An empirical Bayes approach to inferring large-scale gene association networks.** *Bioinformatics* 2005, **21:**754-764.

Table A1-1

Ranking of genes in terms of entropy

	Gene	Entropy
1	*G10*	0.1304
2	*G12*	0.1529
3	*G11*	0.1574
4	*G7*	0.2272
5	*G9*	0.2712
6	*G8*	0.3109
7	*G4*	0.6273
8	*G6*	0.6587
9	*G3*	0.6608
10	*G1*	0.6767
11	*G2*	0.6891
12	*G5*	0.7112

Table A1-2

Ranking of gene pairs in terms of synergy

	Gene 1	Gene 2	Synergy	Entropy
1	*G3*	*G5*	**0.1432**	0.2288
2	*G1*	*G2*	**0.1195**	0.2464
3	*G3*	*G6*	**0.0853**	0.2342
4	*G3*	*G4*	**0.0532**	0.2349
5	*G2*	*G5*	-0.0532	0.4536
6	*G1*	*G5*	-0.0703	0.4581
7	*G2*	*G3*	-0.0727	0.4226
8	*G1*	*G3*	-0.0728	0.4102
9	*G1*	*G6*	-0.0961	0.4315
10	*G2*	*G4*	-0.1045	0.4209

Table A1-3

Ranking of gene pairs in terms of entropy

	Gene 1	Gene 2	Entropy	Synergy
1	*G10*	*G12*	0.0288	-0.7455
2	*G10*	*G11*	0.0296	-0.7418
3	*G7*	*G12*	0.0396	-0.6594
4	*G9*	*G10*	0.0398	-0.6382
5	*G7*	*G10*	0.0421	-0.6845
6	*G7*	*G11*	0.0447	-0.6601
7	*G8*	*G12*	0.0492	-0.5854
8	*G8*	*G10*	0.0498	-0.6085
9	*G8*	*G11*	0.0510	-0.5827
10	*G9*	*G12*	0.0543	-0.6302

Figure legends

Figure A1-1 Network for all healthy samples. Solid lines represent positive correlation and dashed lines represent negative correlation. The intensity of the line corresponds to the magnitude of the correlation.

Figure A1-2 Network for all cancerous samples.

Figure A1-3 Network for all samples (both healthy and cancerous samples combined).

Figure A1-4 Network for all samples, with an additional “outcome” node.

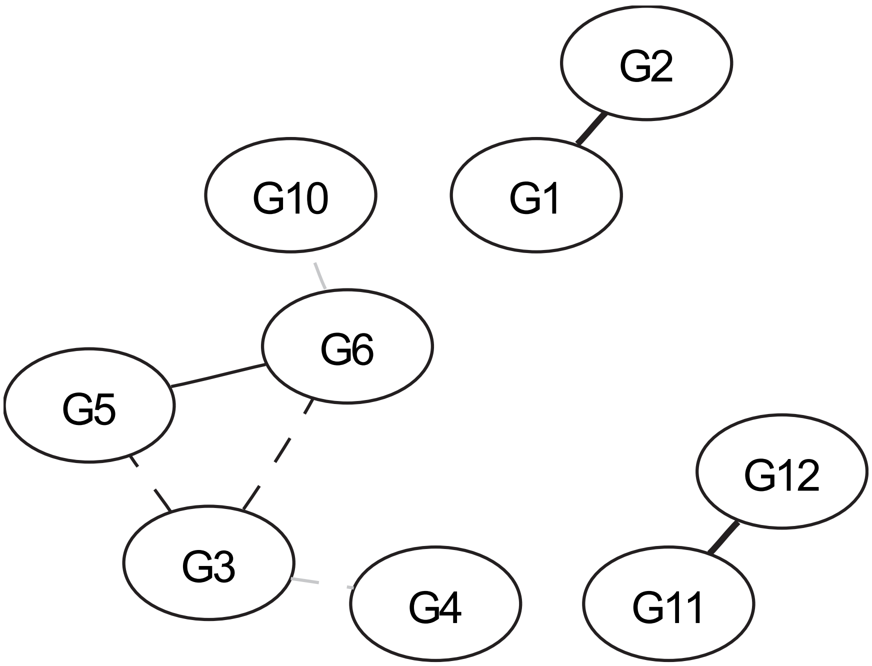

Figure A1-1

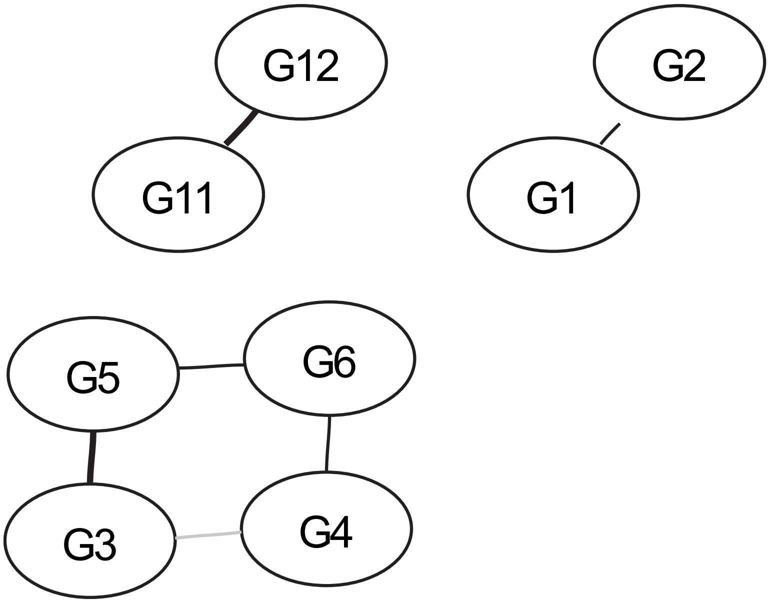

Figure A-2

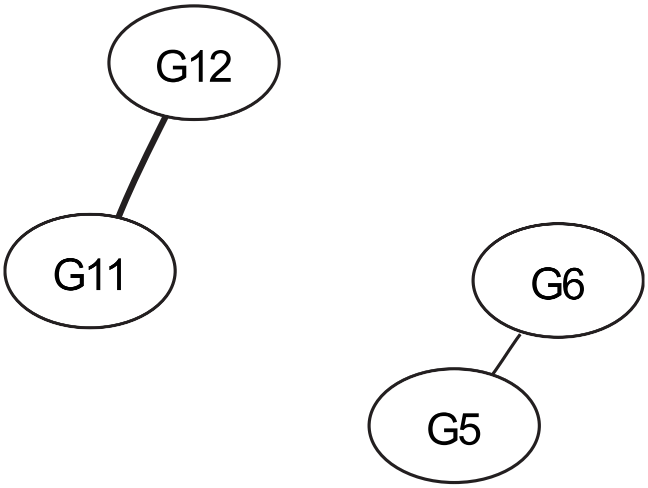

Figure A1-3

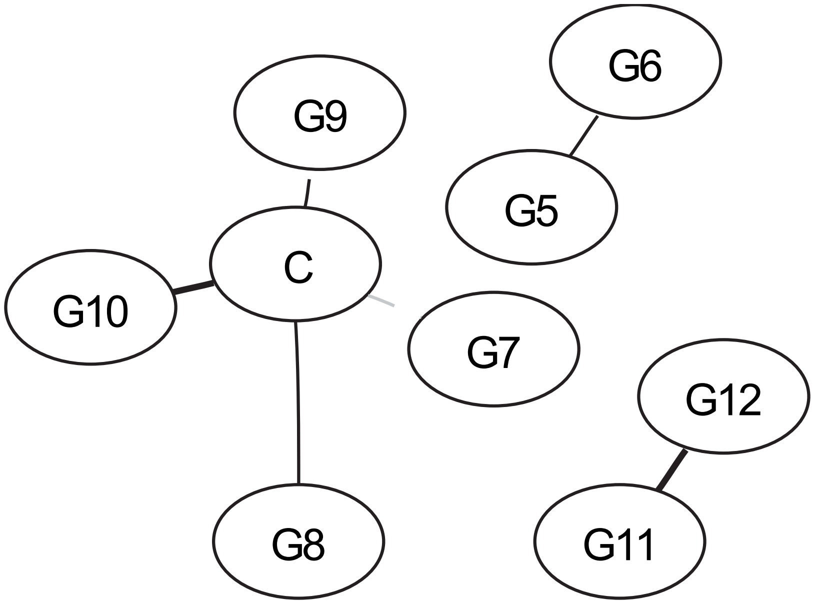

Figure A1-4
