# Supplementary material for: Identification of gene interactions associated with disease from gene expression data using synergy networks
Source: BMC Syst Biol. 2008 Jan 30;2:10. doi: 10.1186/1752-0509-2-10 (PMC2258206; doi:10.1186/1752-0509-2-10)
Supplement: Additional file 2 — Synergy values in validation dataset. Results of applying the synergy network algorithm on an independent dataset used for validation. [file 1752-0509-2-10-S2.pdf]

Additional file 2

Synergy values in validation dataset

The following table shows the highest-synergy gene pairs of the validation data set.

Probe 1	Gene 1	Probe 2	Gene 2	Synergy	Original Data Set Synergy
1786_at	*MERTK*	36780_at	*CLU*	0.3161	0.0334
115_at	*THBS1*	41137_at	*PPP1R12B*	0.2815	0.0350
1005_at	*DUSP1*	33222_at	*FZD7*	0.2424	0.0623
1038_s_at	*IFNGR1*	36780_at	*CLU*	0.2413	0.1102
1450_g_at	*PSMA4*	36780_at	*CLU*	0.2380	0.0819
1042_at	*RARRES1*	41242_at	*UAP1*	0.2337	0.0654
1164_at	*UBCH5*	41191_at	*KIAA0992*	0.2039	0.0460
1107_s_at	*G1P2*	36686_at	*ALDH1A3*	0.2001	0.0742
1009_at	*HINT1*	36577_at	*PLEKHC1*	0.1939	0.2041
1074_at	*RAB1A*	41191_at	*KIAA0992*	0.1885	0.0630

All ten synergy values were found positive in the original data set and all ten were larger than the average synergy (0.0298) over all gene pairs in the dataset, confirming that the synergy values between the two data sets are correlated. As in the original dataset, we performed statistical validation using extreme value Gumbel distribution following 100 permutations. The *P* value of the top-ranked gene pair was found equal to 0.10, which is above any threshold of statistical significance. Therefore, numerical evaluation of synergy from the limited validation dataset is not meaningful, which explains why there is no overlap between the above set of gene pairs and the one found in the original data sets. However, the statistical significance of the top-ranked pairs is still sufficiently high to result in above-average synergy values for all these ten gene pairs when evaluated on the rich original dataset. On the other hand, the synergy values in the validation dataset of the four top-ranked gene pairs shown in Figure 5 were found to be –0.044 (*RBP1*, *EEF1B2*), 0.011 (*PTGDS*, *YWHAQ*), –0.091 (*NME2*, *COX7A1*) and 0.035 (*PTN*, 1514_g_at).

The statistical insignificance of the synergy scores in the validation set is due to the small number of samples, particularly of the healthy samples. In contrast, the sufficiently large original data set yielded extremely low P values ($P < 10^{-15}$ for the top-ranked gene pair and 473 gene pairs with FDR-adjusted $P < 0.05$). Indeed, the Boolean logic that gives rise to the high synergy is preserved in the validation data set (Figure 5), but the small number of samples does not allow for a meaningful numerical evaluation of synergy. As mentioned in the Conclusions of this paper, we hope that high-quality and rich gene expression databases for both diseased and control samples under uniform conditions becomes publicly available, because multivariate analysis of such datasets has the potential of shedding light on pathways responsible for disease.
