# Supplementary material for: Identification of gene interactions associated with disease from gene expression data using synergy networks
Source: BMC Syst Biol. 2008 Jan 30;2:10. doi: 10.1186/1752-0509-2-10 (PMC2258206; doi:10.1186/1752-0509-2-10)
Supplement: Additional file 3 — Software for evaluating entropy and synergy. MATLAB scripts are provided for evaluating conditional entropy and synergy from gene expression data and a corresponding phenotype indicator. [file 1752-0509-2-10-S3.pdf]

Additional file 3

MATLAB Scripts (require MATLAB Bioinformatics & Statistics Toolboxes)

Listing 1 – entropy.m

```
function H = entropy(g, D, c, E)
% Computes the normalized conditional entropy with respect to
phenotype.
%   g - the gene indices.
%   D - the cutoff distance.
%   c - the phenotype indicator, a Nx1 matrix. 1 - Case, 0 - Control.
%   E - the expression data itself, an MxN matrix, M is the # of genes.

K = length(c);     %number of tissues, in our case K=102
q = sum(c)/K;     %Prob(cancer)
Hnorm = -q*log2(q)-(1-q)*log2(1-q);  %so we normalize H later
n = length(g);
F=[];

for i=1:n
    F=[F E(g(i),:)'];  %create a K by n matrix for selected genes
end

dist=pdist(F,'chebychev'); %find all pairwise distances
tree=linkage(dist,'average'); %create the cluster tree
%dendrogram(tree,0); %if we wish to plot the tree
numunder = sum(tree(:,3)<D);%the number of intermediate nodes under D

%ENTROPY EVALUATION
Hcumm = 0;
for iunder = 1:numunder
    cutoff=tree(iunder,3)+0.000001;
    %construct the clusters from the tree
    T=cluster(tree,'cutoff',cutoff,'criterion','distance');
    H=0;
     for i=1:max(T) %max(T) is the number of clusters
         setx = (T==i);
         numc = sum(c(setx)==1);
         numh = sum(c(setx)==0);
         p = numc/(numc+numh);
         if p~=0 && p~=1
             H=H-((p*log2(p)+(1-p)*log2(1-p)))*(numc+numh)/K;
         end
     end
     H=H/Hnorm;
     if iunder<numunder
         Hcumm=Hcumm+H*(tree(iunder+1,3)-tree(iunder,3));
     else
         Hcumm=Hcumm+H*(D-tree(iunder,3));
     end
 end

H=Hcumm/D;
```

Listing 2 – synergy.m

```
function S = synergy(g, D, c, E)
% Computes the normalized synergy of two genes with respect to
% phenotype.
%   g - the gene indices, must be a 1x2 matrix.
%   D - the cutoff distance.
%   c - the phenotype indicator, a Nx1 matrix. 1 - Case, 0 - Control.
%   E - the expression data itself, a MxN matrix, M is the number of
genes.

n = length(g);

if (n ~= 2)
    error('Must supply a 1x2 matrix of genes.');
else
    g1 = g(1);
    g2 = g(2);
    % Compute H(C|G1) normalized by H(C)
    H1 = entropy(g1, D, c, E);
    % Compute H(C|G2) normalized by H(C)
    H2 = entropy(g2, D, c, E);
    % Compute H(C|G1,G2) normalized by H(C)
    H12 = entropy(g, D, c, E);
    % Now compute normalized synergy (all terms are normalized by H(C))
    % Synergy = H(C|G1) + H(C|G2) - H(C|G1,G2) - H(C)
    S = H1 + H2 - H12 - 1;
end
```
